# Optomechanically-induced transparency in parity-time-symmetric microresonators

**DOI:** 10.1038/srep09663

**Published:** 2015-06-12

**Authors:** H. Jing, Şahin K. Özdemir, Z. Geng, Jing Zhang, Xin-You Lü, Bo Peng, Lan Yang, Franco Nori

**Affiliations:** 1 The Key Laboratory of Quantum Optics, Shanghai Institute of Optics and Fine Mechanics, Chinese Academy of Science, Shanghai 201800,China; 2 CEMS, RIKEN, Saitama, 351-0198, Japan; 3 Electrical and Systems Engineering, Washington University, St. Louis, Missouri 63130, U.S.A.; 4 Department of Automation, Tsinghua University, Beijing 100084, China; 5 School of physics, Huazhong University of Science and Technology, Wuhan 430074, China; 6 Physics Department, The University of Michigan, Ann Arbor, MI 48109–1040, U.S.A; 7 Department of Physics, Henan Normal University, Xinxiang 453007, China

## Abstract

Optomechanically-induced transparency (OMIT) and the associated slowing of light provide the basis for storing photons in nanoscale devices. Here we study OMIT in parity-time (*PT*)-symmetric microresonators with a tunable gain-to-loss ratio. This system features a sideband-reversed, non-amplifying transparency , i.e., an inverted-OMIT. When the gain-to-loss ratio is varied, the system exhibits a transition from a *PT*-symmetric phase to a broken-*PT*-symmetric phase. This *PT*-phase transition results in the reversal of the pump and gain dependence of the transmission rates. Moreover, we show that by tuning the pump power at a fixed gain-to-loss ratio, or the gain-to-loss ratio at a fixed pump power, one can switch from slow to fast light and vice versa. These findings provide new tools for controlling light propagation using nanofabricated phononic devices.

Recent advances in steering a macroscopic mechanical object in the deep quantum regime^1^,^2^,^3^ have motivated theoretical studies to understand the physics of photon-phonon interactions in cavity optomechanics (COM), and also led to exciting experimental studies on quantum nanodevices^4^,^5^. In particular, the experimental demonstration of OMIT allows the control of light propagation at room temperature using nano- and micro-mechanical structures^6^,^7^,^8^. The underlying physics of OMIT is formally similar to that of electromagnetically-induced transparency (EIT) in three-level Λ-type atoms^6^,^7^,^8^,^9^,^10^ and its all-optical analogs demonstrated in various physical systems^10^,^11^. The resulting slow-light propagation provides the basis for a wide range of applications^9^. Mechanically-mediated delay (slow-light) and advancement (fast-light) of microwave pulses were also demonstrated in a superconducting nanocircuit^12^,^13^,^14^,^15^. These experimental realizations offer new prospects for on-chip solid-state architectures capable of storing, filtering, or synchronizing optical light propagation.

As a natural extension of single-cavity structures, COM with an auxiliary cavity (compound COM: two cavities) has also attracted intense interest. The interplay between COM interactions and tunable optical tunnelling provides a route for implementing a series of important devices, such as phonon lasers^16^, phononic processors for controlled gate operations between flying (optical) or stationary (phononic) qubits^17^,^18^, and coherent optical wavelength converters^19^,^20^,^21^. Enhanced nonlinearities^22^ and highly-efficient photon-phonon energy transfer^23^,^24^,^25^ are other advantages of the compound COM. These studies were performed with passive (lossy, without optical gain) resonators.

Very recently, an optical system whose behavior is described by *PT*-symmetric Hamiltonians (i.e., the commutator [*H, PT*] = 0)^26^,^27^ was demonstrated in a system of two coupled microresonators, one of which has passive loss and the other has optical gain (active resonator)^28^. Observed features include: real eigenvalues in the *PT*-symmetric regime despite the non-Hermiticity of the Hamiltonian, spontaneous *PT*-symmetry breaking, as well as complex eigenvalues and field localization in the broken *PT*-symmetry regime. Moreover, nonreciprocal light transmission due to enhanced optical nonlinearity in the broken *PT*-symmetry regime was demonstrated^26^. Such a *PT*-symmetric structure provides unique and previously-unattainable control of light and even sound^26^,^27^,^28^,^29^,^30^,^31^,^32^. Manipulating the photon-phonon interactions in such systems opens new regimes for phonon lasing and quantum COM control^33^.

In this paper, we show that a compound COM with *PT*-symmetric microresonators leads to previously unobserved features and provides new capabilities for controlling light transmission in micro- and nano-mechanical systems. Particularly, we show: (i) a gain-induced reversed transparency (inverted-OMIT), i.e. an optical spectral dip between two strongly-amplifying sidebands, which is in contrast to the non-absorptive peak between strongly absorptive sidebands in the conventional passive OMIT; (ii) a reversed pump dependence of the optical transmission rate, which is most significant when the gain and loss are balanced (i.e., optical gain in one subsystem completely compensates the loss in the other); and (iii) a gain-controlled switching from slow (fast) light to fast (slow) light in the *PT*-symmetric (*PT*-breaking) regime, within the OMIT window. These features of the active OMIT enable new applications which are not possible in passive COM.

The inverted-OMIT observed here in an active COM, composed of a passive and an active optical microresonator, is reminiscent of the inverted-EIT observed in all-optical systems, composed of one active and one passive fiber loop^34^. In Ref. 34 a non-amplifying window accompanied with a negative group delay (fast light) was reported. However, our active OMIT, relies on hybrid photon-phonon interactions in a compound COM^6^. In the *PT*-symmetric regime, it provides the first OMIT analog of the optical inverted-EIT^34^. Distinct features of the inverted-OMIT that cannot be observed in the optical inverted-EIT are also revealed in the broken-*PT*-symmetric regime.

## Results

### The active COM system

We consider a system of two coupled whispering-gallery-mode microtoroid resonators^10^,^16^,^35^,^36^. One of the resonators is passive and contains a mechanical mode of frequency *ω*_m_ and an effective mass *m*^16^. We refer to this resonator as the optomechanical resonator. The second resonator is an active resonator which is coupled to the first one through an evanescent field. The coupling strength *J* between the resonators can be tuned by changing the distance between them. As in Ref. 28, the active resonator can be fabricated from Er^3+^-doped silica and can emit photons in the 1550 nm band, when driven by a laser in the 980 nm or 1450 nm bands. The resonators can exchange energies only in the emission band of 1550 nm, so the gain photons can tunnel through the air gap between the resonators and provide a gain *κ* to compensate the optical loss γ in the passive resonator^28^.

**Figure 1:**
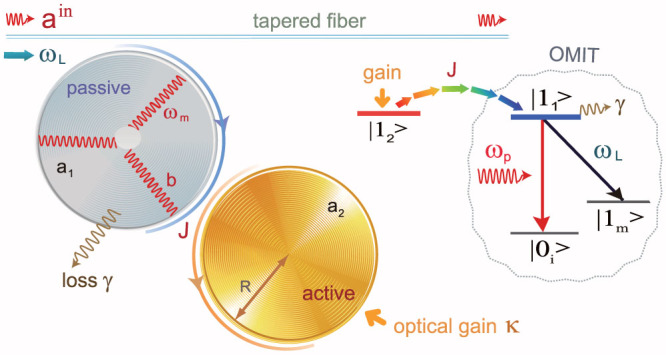
OMIT in active-passive-coupled micro-resonators, with a tunable gain-loss ratio. The similarity of the passive OMIT and the three-level EIT is well-known^6^; in parallel, the active OMIT provides a COM analog of the optical inverted-EIT^34^ (see the energy levels with an input gain). Here |0*_i_*〉 = |*n*_1_, *n*_2_, *n*_m_〉, |1_1_〉 = |*n*_1_ + 1, *n*_2_, *n*_m_〉, |1_2_〉 = |*n*_1_, *n*_2_ + 1, *n*_m_〉, |1_m_〉 = |*n*_1_, *n*_2_, *n*_m_ + 1〉, while *n*_1,2_ and *n*_m_ denote the number of photons and phonons, respectively.

Tuning the gain-to-loss ratio, while keeping *J* fixed, leads to two remarkably distinct regimes, i.e. broken- and unbroken-*PT* -symmetry regimes, that are characterized by distinct normal mode-splitting and linewidths^28^,^33^. Our aim here is to study OMIT in these two distinct regimes, focusing on the role of *κ*/γ. To this end, as in the conventional OMIT^6^, both a pump laser of frequency *ω*_L_ and a weak probe light of frequency *ω_p_* are applied (see Fig. 1). The field amplitudes of the pump and probe are given by *E*_L_ = (2*P*_L_γ/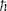
*ω*_L_)^1/2^, *ε_p_* = (2*P*_in_γ/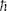
*ω_p_*)^1/2^, where *P*_L_ and *P*_in_ are the pump and probe powers.

The Hamiltonian of this three-mode COM system can be written as
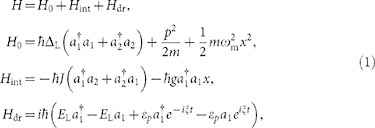
where *a*_1_ and *a*_2_ denote the annihilation operators of the bosonic fields in the microresonators with resonance frequency *ω_c_* and radius *R*, *g* = *ω_c_*/*R* is the COM coupling rate, *x* = *x*_0_(*b* + *b*^†^) is the mechanical position operator, *x_0_* = [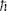/(2*mω*_m_)]^1/2^, and *b* corresponds to the annihilation operator for the phonon mode. The pump-resonator, probe-resonator, and probe-pump frequency detunings are, respectively, denoted by
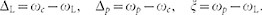


The Heisenberg equations of motion (EOM) of this compound system are (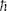 = 1)
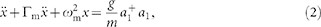

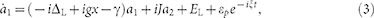

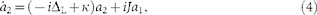
where Γ_m_ is the mechanical damping rate. The optical or mechanical gain and damping terms are added phenomenologically into the EOM^24^,^33^,^35^; they can also be incorporated into Eq. (1), resulting in a non-Hermitian Hamiltonian^26^,^27^,^28^,^29^,^30^,^31^,^32^,^33^,^36^. We note that *κ* < 0 in Eq. (4) corresponds to a passive-passive COM; thus *κ*/γ < 0 and *κ*/γ > 0 define, respectively, a passive-passive COM and a passive-active COM.

The steady-state values of the dynamical variables are
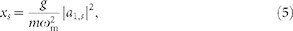

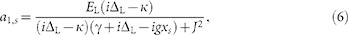

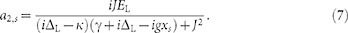


For Δ_L_ = 0, by choosing *J*^2^ = *k*γ or *κ*/γ → 1 for *J* = γ, one can identify a gain-induced transition from the linear to the nonlinear regime that significantly enhances COM interactions^33^. Here we focus on the effects of the gain-loss balance on the OMIT and the associated optical group delay, which, to our knowledge, has not been studied previously.

We proceed by expanding each operator as the sum of its steady-state value and a small fluctuation around that value, i.e. *a*_1_ = *a*_1,*s*_ + *δa*_1_, *a*_2_ = *a*_2,*s*_ + *δa*_2_, *x* = *x_s_* + *δx*. After eliminating the steady-state values, we obtain the linearized EOM, which can be solved using the ansatz (see the Method)
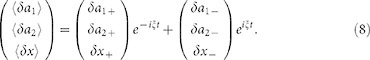
The optical fluctuation in the optomechanical resonator 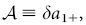 the quantity of interest here, is
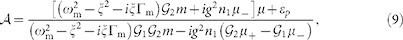
where *n*_1_ = |*a*_1,*s*_|^2^ is the intracavity photon number of the passive resonator, *µ*_±_ = −*κ*−*iξ* ± *i*Δ_L_, and
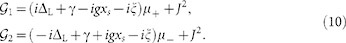
The expectation value of the output field can then be obtained by using the standard input-output relation, i.e. 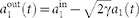, where 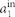 and 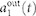 are the input and output field operators. Then the optical transmission rate *η*(*ω_p_*) (i.e., the amplitude square of the ratio of the output field amplitude to the input probe field amplitude, 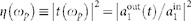) is
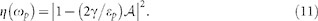
We computed Eq. (11) with experimentally-accessible values of the system parameters^28^ to better understand the behavior of the COM in the presence of gain and loss. These parameters are *R* = 34.5 *µ*m, *ω_c_* = 1.93 × 10^5^ GHz, *ω*_m_ = 2*π* × 23.4 MHz, *m* = 5 × 10^−11^ kg, γ = 6.43 MHz and Γ_m_ = 2.4 × 10^5^ Hz. The quality factors of the optical mode and the mechanical mode in the passive resonator are *Q_c_* = 3 × 10^7^ and 2*Q*_m_/*Q_c_* = 10^−5^, respectively. Also Δ_L_ = *ω*_m_, and thus Δ*_p_* ≡ *ω_p_* − *ω_c_* = *ξ* − *ω*_m_. Now we discuss how the gain-loss ratio *κ*/γ, the coupling strength *J*, and the pump power *P*_L_ affect the OMIT. Note that *κ*/γ and *J* are the tunable system parameters that allow one to operate the system in the broken- or unbroken-*PT* regimes.

### Reversed-gain dependence

**Figure 2:**
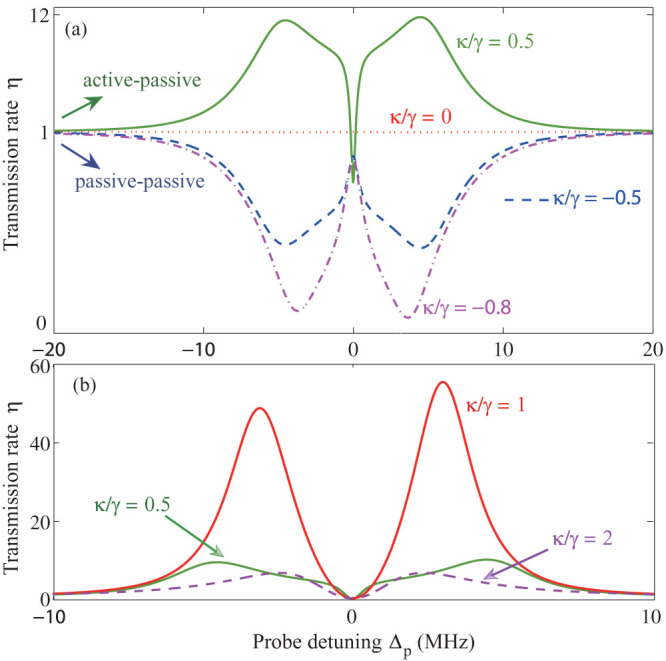
The transmission rate *η* of the probe light versus the optical detuning Δ*_p_* = *ω_p_* − *ω_c_*, for different values of the gain-loss ratio. (a) Around the transient point *κ*/γ = 0. (b) Around the balanced point *κ*/γ = 1. The optical tunnelling rate and the pump power are fixed as *J*/γ = 1 and *P*_L_ = 10 *µ*W, respectively. Note that although it is not seen clearly in (a), the case *κ*/γ = 0 (red dotted line) has OMIT features of sideband dips and a resonance peak (see Fig. 3).

Figure 2 depicts the effect of *κ*/γ on the optical transmission rate. By introducing gain into the second microresonator, one can tune the system to transit from a conventional OMIT profile, quantified by a transparency window and two sideband dips, to the inverted-OMIT profile, quantified by a transmission dip and two sideband peaks (see Fig. 2a).

Increasing the loss ratio *κ*/γ < 0 in the passive-passive COM leads to shallower sidebands. When the amount of gain provided to the second resonator supersedes its loss and the resonator becomes an active one (amplifying resonator), increasing *κ*/γ > 0 helps to increase the heights of the sideband peaks until *κ*/γ = 1, where *η* at sidebands is maximized. Increasing the gain further leads to the suppression of both the sideband peaks and the on-resonance (Δ*_p_* = 0) transmission (Fig. 2b). This is in stark contrast with the observation of monotonically-increasing sideband peaks in the all-optical EIT system of Ref. 34.

This can be intuitively explained as follows. Under the condition of *J*/γ = 1, the system is in the *PT*-symmetric phase for *κ*/γ < 1, whereas it is in the broken-*PT* phase for *κ*/γ > 1. Thus, for *κ*/γ < 1, the provided gain compensates a portion of the losses, which effectively reduces the loss in the system and hence increases *η*^28^. Increasing the gain above the phase transition point *κ*/γ = 1 puts the system in the broken-*PT* phase, with a localized net loss in the passive resonator^28^ (i.e., the field intensity in the passive resonator is significantly decreased) which reduces the strength of the COM interactions and hence the value of *η*. The reduction of the transmission by increasing the gain provides a signature of the *PT*-breaking regime, and it is very similar to a recent experiment with two coupled-resonators where it was shown that increasing (decreasing) the loss of one of the resonators above (below) a critical level increases (decreases) the intracavity field intensity of the other, enhancing (suppressing) transmission^36^. Note that increasing (decreasing) loss is similar to decreasing (increasing) gain. We conclude here that only in the *PT*-symmetric regime (*κ*/γ < 1, with *J*/γ = 1), the active OMIT can be viewed as an analog of the optical inverted-EIT^34^.

**Figure 3:**
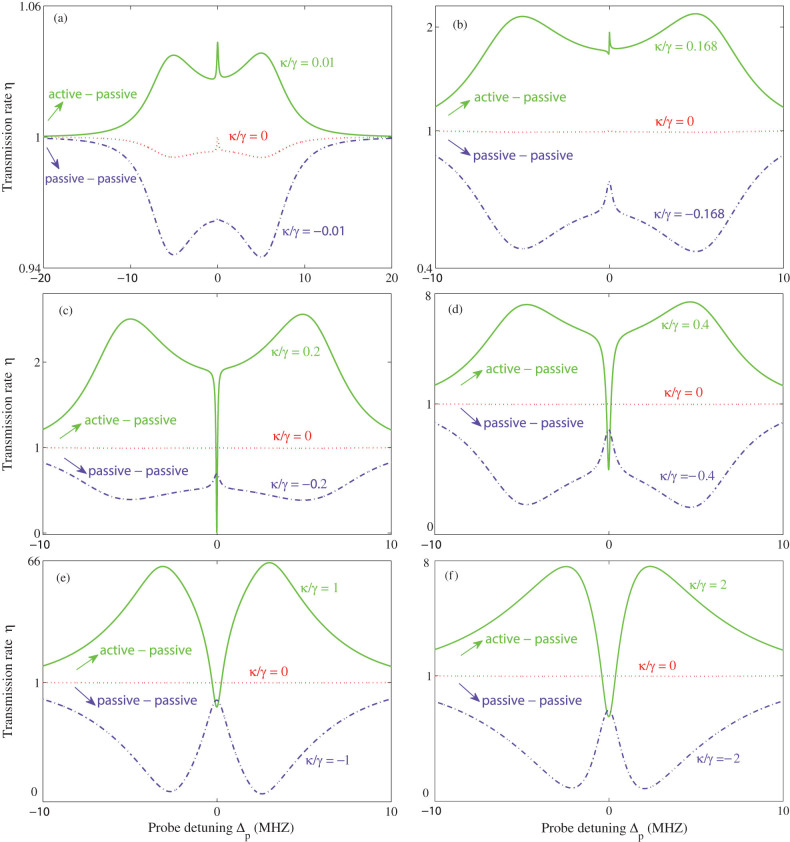
The transmission rate *η* of the probe light in the active-passive system. The relevant parameters are taken as *J*/γ = 1 and *P*_L_ = 10 *µ*W.

Figure 3 provides the fine features in the transmission rate, by numerically changed *κ*/γ > 0 by very small steps. One interesting observation in Fig. 3 is that when *κ* = 0, the second resonator has neither gain nor loss, and there still exists OMIT-like spectrum, i.e. small fluctuations around *η* ~ 1. In this case, the coupled-resonator system is still a passive-OMIT because one resonator is lossy while the other has neither loss nor gain. For *κ*/γ = 0.01, we have a small resonance peak (see Fig. 3a). When the gain is increased, this peak tends to disappear (see e.g. Fig. 3b) and then evolves into a dip, e.g. *η* ~ 0 for *κ*/γ = 0.2 (see Fig. 3c). This dip can also be manipulated by increasing the gain further [see Figs. 3(d–f)]. These results imply that one can tune the system from passive-OMIT to active-OMIT, or vice versa, by varying the gain-to-loss ratio *κ*/γ. Such transient behaviors have not been revealed previously.

### Reversed-pump dependence

**Figure 4:**
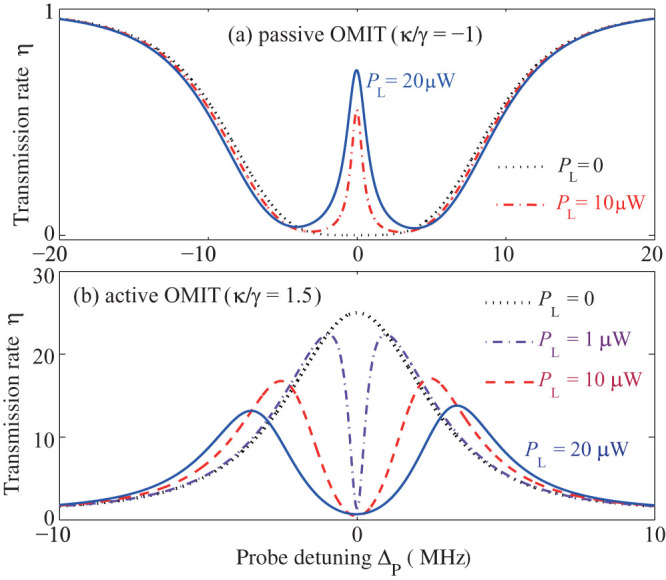
Diagrams of the transmission rate of the probe light for different systems. (a) The passive-passive COM system, with *κ*/γ = −1. (b) The active-passive COM system, with *κ*/γ = 1.5 (similar results with e.g. *κ*/γ = 0.5 or *κ*/γ = 1 are not shown here).

For the passive-passive COM, the transmission rate and the width of the OMIT window increase with increasing pump power *P*_L_^6^,^24^ (see also Fig. 4a). For the passive-active COM, where we observe the inverted-OMIT, increasing the pump power *P*_L_ leads to a significant decrease of the sideband amplifications (Fig. 4b). Here the pump power dependence of the OMIT profile is shown for *κ*/γ = 1.5 (in the *PT*-breaking regime). We have also performed our calculations for *κ*/γ = 1 and *κ*/γ = 0.5, and similarly found that in these cases the sideband amplifications are also reduced as the pump is increased from *P*_L_ = 10 *µ*W to 20 *µ*W (not shown here). Nevertheless, the sideband amplification always reaches its maximum value at the gain-loss balance (see also Fig. 2b). We note that the counterintuitive effect of reversed pump dependence was also previously demonstrated in coupled optical systems (i.e., no phonon mode was involved) operating at the exceptional point^36^,^37^.

In addition, we have also studied the effect of the mechanical damping on the profiles of the conventional and inverted OMIT at different values of the gain-to-loss ratio. We confirmed that the profiles of both the conventional and the inverted OMIT are strongly affected (i.e., tend to disappear) by increasing the mechanical damping. This highlights the key role of the mechanical mode in observing OMIT-like phenomena.

### *PT*-breaking fast light

**Figure 5:**
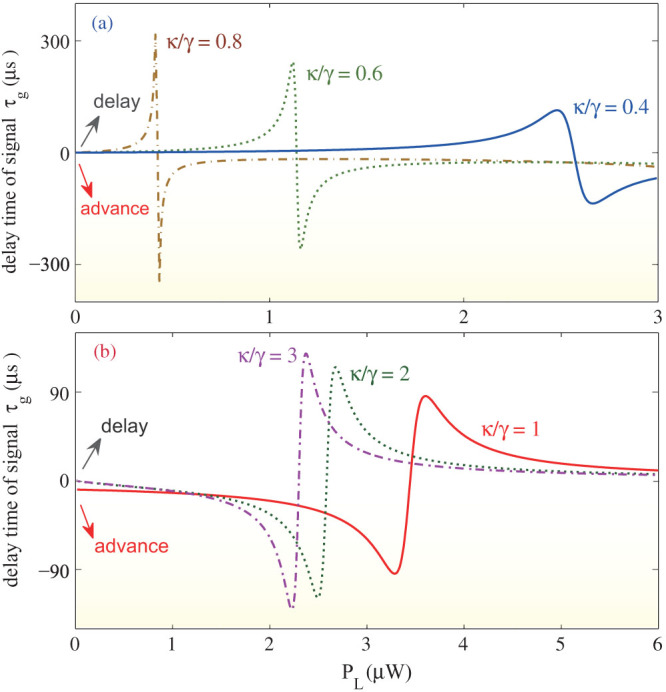
The delay or advance of the probe light in active-passive-coupled resonators, characterized by positive or negative group delays. (a) The *PT*-symmetric regime with *κ*/γ < 1. (b) The *PT*-breaking regime with *κ*/γ ≥ 1. In these calculations we have taken Δ*_p_* = 0, *J*/γ = 1.

The light transmitted in an EIT window experiences a dramatic reduction in its group velocity due to the rapid variation of the refractive index within the EIT window, and this is true also for the light transmitted in the OMIT window in a conventional passive optomechanical resonator^6^. Specifically, the optical group delay of the transmitted light is given by
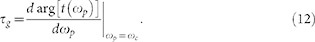
We have confirmed that OMIT in the passive-passive COM leads only to the slowing (i.e., positive group delay: *τ_g_* > 0) of the transmitted light, and that when the coupling *J* between the resonators is weak the reduction in the group velocity approaches to that experienced in a single passive resonator^6^,^24^. In contrast, in the active-passive COM, one can tune the system to switch from slow to fast light, or vice versa, by controlling *P*_L_ or *κ*/γ, such that the COM experiences the *PT*-phase transition (Fig. 5).

In the regime *κ*/γ < 1, as *P*_L_ is increased from zero, the system first enters into the slow-light regime (*τ_g_* > 0), and *τ_g_* increases until its peak value. Then it decreases, reaching *τ_g_* = 0, at a critical value of *P*_L_ (Fig. 5a). The higher is the *κ*/γ, the sharper is the decrease. Increasing *P*_L_ beyond this critical value completes the transition from slow to fast light and *τ_g_* becomes negative (*τ_g_* < 0). After this transition, the advancement of the pulse increases with increasing *P*_L_ until it reaches its maximum value (more negative *τ_g_*). Beyond this point, a further increase in *P*_L_, again, pushes *τ_g_* closer to zero.

In the regime *κ*/γ > 1, increasing *P*_L_ from zero first pushes the system into the fast-light regime and increases the advancement of the pulse (*τ_g_* < 0) until the maximum advance is reached (Fig. 5b). After this point, the advance decreases with increasing *P*_L_ and finally *τ_g_* becomes positive, implying a transition to slow light. If *P*_L_ is further increased, *τ_g_* first increases until its peak value, and then decreases approaching *τ_g_* = 0.

The *P*_L_ value required to observe the transition from slow-to-fast light (when *κ*/γ < 1) or from fast-to-slow light (when *κ*/γ > 1) depends on the gain-to-loss ratio *κ*/γ if the coupling strength *J* is fixed (Fig. 5). This implies that, when *P*_L_ is kept fixed, one can also drive the system from slow-to-fast or fast-to-slow light regimes by tuning *κ*/γ. A simple picture can be given for this numerically-revealed feature: for Δ_L_ ~ 0, *ξ* ~ 0, we simply have 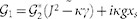 which is minimized for *J*^2^ = *κ*γ, or *κ*/γ = 1, *J*/γ = 1; therefore, in the vicinity of the gain-loss balance, the denominator of *A* is a real number, and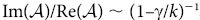, i.e. having reverse signs for *κ*/γ > 1 or *κ*/γ < 1. Correspondingly, arg(*A*) or arg[*t*(*ω_p_*)] and hence its first-order derivative *τ_g_* ~ (γ/*κ* −1) (for *J*/*κ* = 1). Clearly, the sign of *τ_g_* can be reversed by tuning from the *PT*-symmetric regime (with *κ*/γ < 1) to the broken-*PT* regime (with *κ*/γ > 1). We note that the appearance of the fast light in the *PT*-breaking regime, where the gain becomes to exceed the loss, is reminiscent of that observed in a gain-assisted or inverted medium^38^.

**Figure 6:**
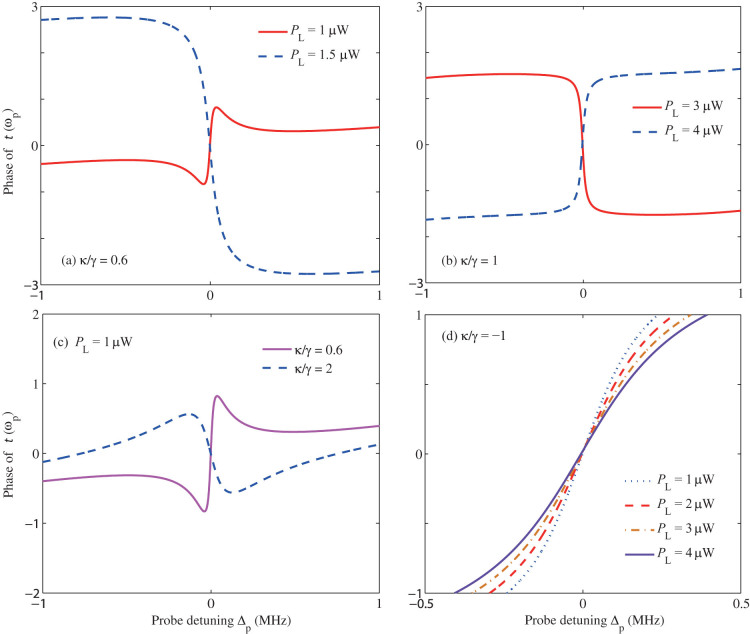
The phase of the transmission amplitude *t*(*ω_p_*) with different parameters. (a-b) With different values of pump power *P*_L_. (c) With different values of gain-to-loss ratio *κ*/γ. The specific values of *P*_L_ and *κ*/γ are taken from Fig. 5, corresponding to the slow and fast light regimes. For comparison, the results for the passive-passive COM system are also plotted in (d) with *κ*/γ = −1.

In order to better visualize and understand how the switching from the slow-to-fast light and vice versa takes place, when the gain-to-loss ratio *κ*/γ is tuned at a fixed-pump power *P*_L_, or when *P*_L_ is tuned at a fixed value of *κ*/γ, we present the phase of the transmission function *t*(*ω_p_*) in Figs. 6(a–c). For this purpose, we choose the values of *P*_L_ and *κ*/γ from Fig. 5, where their effects on the optical group velocity *τ_g_* were presented. These calculations clearly show that, near the resonance point (*δ_p_* = 0), the slope of the curves can be tuned from positive to negative or vice versa, by tuning *P*_L_ or *κ*/γ, which agrees well with the slow-fast light transitions (see Fig. 5). In sharp contrast, Fig. 6d shows that for the passive-passive COM (e.g., *κ*/γ = −1), no such type of sign reversal can be observed for the slope of the phase curves, corresponding to the fact that only the slow light can exist in that specific situation.

## Discussion

In conclusion, we have studied the optomechanically-induced-transparency (OMIT) in *PT*-symmetric coupled microresonators with a tunable gain-loss ratio. In contrast to the conventional OMIT in passive resonators (a transparency peak arising in the otherwise strong absorptive spectral region), the active OMIT in *PT*-symmetric resonators features an inverted spectrum, with a transparency dip between two sideband peaks, providing a COM analog of the all-optical inverted-EIT^34^. For this active-OMIT system, the counterintuitive effects of gain- or pump-induced suppression of the optical transmission rate are revealed. In particular, the transition from slow-to-fast regimes by tuning the gain-to-loss ratio or the pump power is also demonstrated. The possibility of observing the *PT*-symmetric fast light, by tuning the gain-to-loss ratio of the coupled microresonators^28^, has not studied previously. These exotic features of OMIT in *PT*-symmetric resonators greatly widens the range of applications of integrated COM devices for controlling and engineering optical photons. In addition, our work can be extended to study e.g. the OMIT in a quasi-*PT* system^36^, the OMIT cooling of mechanical motion^39^,^40^, the active-OMIT with two mechanical modes^19^, or the gain-assisted nonlinear OMIT^41^,^42^,^43^.

## Methods

### Derivation of the optical transmission rate

Taking the expectation of each operator given in Eqs. (2)–(4), we find the linearized Heisenberg equations as
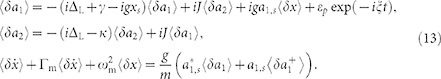
which can be transformed into the following form, by applying the ansatz given in Eq. (8),
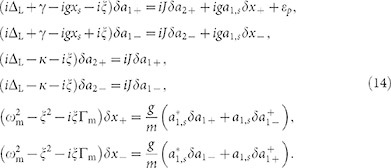
Solving these algebraic equations leads to
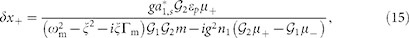

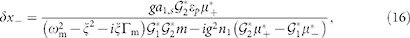

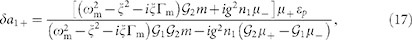

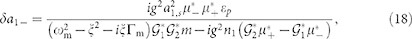

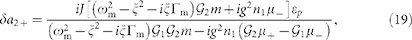

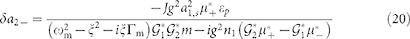
where we have used *n_i_* = |*a_i_*_,*s*_|^2^ (*i* = 1, 2) and
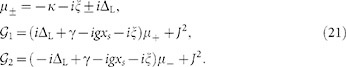
The expectation value 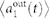 of the output field 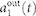 can be calculated using the standard input-output relation 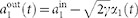, where 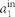 and 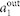 are the input and output field operators, and
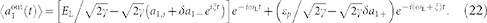
Hence, the transmission rate of the probe field can be written as *η* = |*t*(*ω_p_*)|^2^, where *t*(*ω_p_*) is the ratio of the output field amplitude to the input field amplitude at the probe frequency
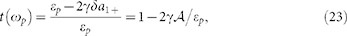
where *A* ≡ *δa*_1+_ is given in Eq. (9). In order to receive some analytical estimations, we take *ω*_m_/*ω_c_* ~ 0, Δ_L,*p*_ ~ 0, which leads to *µ*_±_ ~ −*κ*, 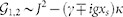. For *x_s_* ~ 0, we have
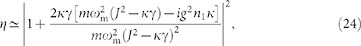
i.e. *η* ~ (*J*^2^ − *κ*γ)^−2^ or *η* ~ (1 − *κ*/γ)^−2^ (for a fixed value of *J*/γ = 1). This indicates that the transmission rate *η* tends to be maximized as the gain-to-loss ratio approaches one, that is *κ*/γ = 1, which was confirmed by our numerical calculations (see Fig. 2b).
