# Author Correction: Optomechanically-induced transparency in parity-time-symmetric microresonators

**DOI:** 10.1038/s41598-022-25159-5

**Published:** 2022-12-02

**Authors:** H. Jing, Şahin K. Özdemir, Z. Geng, Jing Zhang, Xin-You Lü, Bo Peng, Lan Yang, Franco Nori

**Affiliations:** 1 The Key Laboratory of Quantum Optics, Shanghai Institute of Optics and Fine Mechanics, Chinese Academy of Science, Shanghai, 201800 China; 2 CEMS, RIKEN, Saitama, 351-0198 Japan; 3 Electrical and Systems Engineering, Washington University, St. Louis, Missouri 63130 USA; 4 Department of Automation, Tsinghua University, Beijing, 100084 China; 5 School of Physics, Huazhong University of Science and Technology, Wuhan, 430074 China; 6 Physics Department, The University of Michigan, Ann Arbor, MI 48109–1040 USA; 7 Department of Physics, Henan Normal University, Xinxiang, 453007 China

Correction to: *Scientific Reports* https://doi.org/10.1038/srep09663, published online 12 June 2015

In the original version of this Article, Franco Nori is incorrectly affiliated with ‘Electrical and Systems Engineering, Washington University, Missouri, 63130, St. Louis, U.S.A.’ The correct affiliations are listed below.

CEMS, RIKEN, Saitama, 351-0198, Japan

Physics Department, The University of Michigan, Ann Arbor, MI, 48109–1040, U.S.A.

